# Supplementary material for: Self-management for chronic widespread pain including fibromyalgia: A systematic review and meta-analysis
Source: PLoS One. 2021 Jul 16;16(7):e0254642. doi: 10.1371/journal.pone.0254642 (PMC8284796; doi:10.1371/journal.pone.0254642)
Supplement: S1 File — (PDF) [file pone.0254642.s001.pdf]

SELF-MANAGEMENT INTERVENTIONS FOR CHRONIC WIDESPREAD PAIN INCLUDING FIBROMYALGIA

ADMINISTRATION INFORMATION

Registration

The protocol will be registered with the International Prospective Register of Systematic Reviews (PROSPERO)

Authors:

Contact

Corresponding author:
Dr Adam Geraghty, A.W.Geraghty@soton.ac.uk
Primary Care and Population Sciences
Faculty of Medicine
University of Southampton
Aldermoor Health Centre
Southampton
SO16 5ST

Dr Emma Maund, University of Southampton, E.T.Maund@soton.ac.uk
Professor Michael Moore, University of Southampton, M.V.Moore@soton.ac.uk
Dr Hazel Everitt, University of Southampton, H.A.Everitt@soton.ac.uk
Dr Miriam Santer. University of Southampton, M.Santer@soton.ac.uk
Professor Paul Little, University of Southampton, P.Little@soton.ac.uk
Dr Beth Stuart, University of Southampton, bls1@soton.ac.uk
Professor Tamar Pincus, t.pincus@rhul.ac.uk
Dr Cathy Price, Solent NHS Trust, cathy.price@nhs.net
Dr David Newell, University of Southampton, D.Newell@soton.ac.uk
Rachel West, University of Southampton.

Support

Sources

This systematic review is funded by the UK National Institute for Health Research (NIHR) Research Capability Fund, through the Solent NHS Trust R&D Group. The NIHR and Solent NHS Trust had no involvement in the design of the review protocol, and will have no input in data collection, analyses, interpretation, writing up or publication of the review.

INTRODUCTION

Rationale

Chronic Widespread Pain (CWP, including fibromyalgia) has a reported prevalence of up to 15% in the general population (1) causing substantial functional impairment and psychological distress. Although referrals to secondary care and specialist services are common, CWP is primarily managed in primary care (2). The treatment options available to General Practitioners (GPs) are limited. Chronic opiate prescription is common for patients with CWP, however, with growing concerns about opioid dependence (3) in addition to unique pathophysiologic characteristics of people with CWP (4) alternatives to long-term opiate use are necessary. Gabapentinoids are increasingly prescribed as alternatives to opioids, yet their utility is questionable due to a substantial side-effect profile and limited evidence for their effectiveness in CWP (5). The limitations of pharmacotherapy highlight the need for accessible non-pharmacological interventions.

The British Pain Society recently developed a care pathway for CWP (6). The pathway acknowledges the importance of supporting those with CWP to effectively self-manage, enabling them to recover and maintain quality of life, as well as reducing dependence on stretched services (6). Self-management interventions commonly have the broad aim of improving a person's health status through teaching skills to effectively manage a specific condition, often including behavioural, emotional and medical domains (7, 8). Increasing the availability of effective, accessible self-management interventions may substantially improve the primary care management of CWP, and ensure secondary care services can focus on complex cases where alternative approaches may be more appropriate.

Previous systematic reviews of broad self-management in this area have focused on chronic pain in general, grouping CWP with more specific musculoskeletal conditions such as low

back pain, knee pain, shoulder pain and osteoarthritis (7, 9, 10). To inform the development and use of self-management interventions accounting for CWP's unique characteristics and their impact (e.g. fatigue and psychological morbidity), a specific CWP focus is necessary. Review evidence suggests moderate effects for single component interventions in CWP such as exercise (11), and limited effects of single psychotherapeutic approaches including guided imagery (12) and CBT (13). However, Sarzi-Puttini et al (12) suggest a multi-dimensional approach may be critical in CWP (14, 15), and this is likely to apply to self-management, particularly when considering interventions for application in primary care.

Objectives

To systematically review randomised controlled trials of self-management interventions specifically targeting CWP including fibromyalgia. A secondary aim is to explore the effect of delivery modality on the effectiveness of the interventions.

METHODS

Eligibility criteria

Population

Adult patients receiving community, primary or secondary care for chronic widespread pain or fibromyalgia as defined by study authors (diagnostic criteria used will be extracted and documented in the review)

We will include studies in patients with mixed diagnoses (e.g. osteoarthritis, fibromyalgia), provided data are reported separately for patients with our condition of interest.

Intervention

Any self-management intervention that fulfils all of the following criteria (adapted from Miles et al., (7)):

i) has the broad goal of improving participants' health status or quality of life with scope for improvement in patients managing their own health.

ii) aims to increase participants' skills and knowledge and to enable participants to deploy these enhanced skills in aspects of their lives beyond the intervention

iii) is directed at patients

iv) is multicomponent e.g. relaxation plus exercise. Interventions focusing on one single active component will be excluded e.g. exercise or relaxation, or biofeedback alone

> From Miles et al. (7):
>
> "The programme had to contain at least two components from the following five groups agreed by our steering group: psychological (including behavioural or cognitive therapy), mind–body therapies (MBT) (including such as relaxation, meditation or guided imagery), physical activity (any form of exercise), lifestyle (such as dietary advice and sleep management) and medical education (such as understanding their condition and how to take medication effectively)." p775

Comparator

- Placebo
- Waiting list control
- Usual care
- One self-management intervention compared to another active intervention

Outcome

Primary outcomes:

- Physical function/disability
- Pain

Secondary outcomes:

- Medication usage
- Disease specific measures e.g. Fibromyalgia Impact Questionnaire (FIQ)
- Fatigue
- Global health measures
- Quality of life
- Mental health/ psychological wellbeing
- Healthcare utilisation

- Harms

Study design

Randomised controlled trials

Information sources

We will search the following electronic databases from inception: Cochrane Central Register of Controlled Trials (CENTRAL), MEDLINE (Ovid), Embase (Ovid), PsycINFO (EBSCO*host*), and the WHO International Clinical Trials Registry platform. The following supplemental searches will be performed: reference list checking, and contact with experts.

Search strategy

We will create database specific search strategies using subject headings and text words related to CWP and fibromyalgia and self-management interventions, and database specific RCT filters. The MEDLINE strategy will be developed first, and peer reviewed by a Medical Librarian. After the MEDLINE strategy is finalised, it will be adapted to the syntax and subject headings of the other databases. There will be no language restrictions.

Study records

Data management

Titles and abstracts of all articles returned by electronic databases searches and supplemental searches will be saved in an Endnote library. Duplicate titles, where the same articles have been returned by more than one source, will be removed prior to screening.

Selection process

Screening will be performed in Covidence. Two reviewers (EM and AG) will independently screen all titles and abstracts yielded by the search against the inclusion criteria. We will obtain full papers for all titles and abstracts that appear to meet the inclusion criteria or where there is uncertainty. Two reviewers (EM and AG) will then independently assess whether these full papers meet the inclusion criteria. Disagreements will be resolved by discussion, and where applicable, arbitration by a third reviewer (MS or HE). We will record the number of papers at each stage of the process and the reasons for their exclusion.

Data collection process

We will perform data extraction into a pre-piloted Excel data extraction form. Data extracted will include: patient characteristics (e.g. age, sex, diagnostic criteria used, duration of illness, comorbidities); and elements of the Template for Intervention and Replication (TIDierR) checklist (16) including funding source. This includes physical or informational materials used in the intervention.

Risk of bias in individual studies

Risk of bias assessment will be performed by one reviewer and checked by a second. Any disagreements will be resolved by discussion, and where applicable, arbitration by a third reviewer (HE or MS). We will use the Cochrane Risk of Bias tool in accordance with guidance from the Cochrane handbook (17).

Data synthesis

We will present information in text and tables to summarise the characteristics and findings of the included studies. If studies are heterogeneous in terms of intervention and comparator, we will use a narrative approach to explore the relationship and findings both within and between the included studies.

If studies, based on clinical opinion, are sufficiently homogeneous in terms of intervention and comparator, we will conduct a meta-analysis using a random effects model. Data for cluster randomised will be treated according to the methods described in the Cochrane Handbook for Systematic Reviews of Interventions (17). For cross-over trials, we will only use the data from the first period. For dichotomous data, we will calculate risk ratios with 95% confidence intervals (CI). For continuous outcomes, we will calculate mean differences with 95% CI, or standardised mean differences with 95% if different measurement scales are used. When there are missing data, we will attempt to contact the original authors of the study to obtain the relevant missing data. Statistical heterogeneity will be tested using the Chi^2 test (significance level: 0.1) and I^2 statistic. If high levels of heterogeneity among the trials exist ($I^2 \geq 50\%$ or $P < 0.1$) we will explore sources of heterogeneity using subgroup analysis or sensitivity analysis.

Analysis of subgroups or subsets

If sufficient data is available:

- Sensitivity analyses will be conducted to explore whether results would differ if studies at high risk of bias were excluded
- Subgroup analysis will be performed for mode of delivery (e.g. individual versus group versus self-directed).
- Subgroup analysis will be performed for diagnosis of fibromyalgia versus studies of CWP more generally
- Subgroup analysis will be performed for type of control condition
- Subgroup analysis will be performed to explore whether results would differ depending on the components included in the interventions (e.g. interventions including psychoeducation and/or exercise)

Reporting of review

Reporting of the full systematic review will follow the Preferred reporting items for systematic reviews and meta-analyses (PRISMA) guidelines (18).

REFERENCES

1. Mansfield KE, Sim J, Jordan JL, Jordan KP. A systematic review and meta-analysis of the prevalence of chronic widespread pain in the general population. Pain. 2016;157:55-64.

2. Sullivan MD, Turner JA, Romano J. Chronic pain in primary care. Identification and management of psychosocial factors. J Fam Pract. 1991;32:193-199.

3. Crofford LJ. Adverse effects of chronic opioid therapy for chronic musculoskeletal pain. Nat Rev Rheumatol. 2010;6:191-197.

4. Painter JT, Crofford LJ. Chronic opioid use in fibromyalgia syndrome: a clinical review. J Clin Rheumatol. 2013;19:72-77.

5. Goodman CW, Brett AS. Gabapentin and Pregabalin for Pain - Is Increased Prescribing a Cause for Concern. N Engl J Med. 2017;377:411-414.

6. Lee J, Ellis B, Price C, Baranowski AP. Chronic widespread pain, including fibromyalgia: a pathway for care developed by the British Pain Society. Br J Anaesth. 2014;112:16-24.

7. Miles CL, Pincus T, Carnes D et al. Can we identify how programmes aimed at promoting self-management in musculoskeletal pain work and who benefits? A systematic review of sub-group analysis within RCTs. Eur J Pain. 2011;15:775.e1-11.

8. Lorig KR, Holman H. Self-management education: history, definition, outcomes, and mechanisms. Ann Behav Med. 2003;26:1-7.

9. Du S, Yuan C, Xiao X, Chu J, Qiu Y, Qian H. Self-management programs for chronic musculoskeletal pain conditions: a systematic review and meta-analysis. Patient Educ Couns. 2011;85:e299-310.

10. Carnes D, Homer KE, Miles CL et al. Effective delivery styles and content for self-management interventions for chronic musculoskeletal pain: a systematic literature review. Clin J Pain. 2012;28:344-354.

11. Bidonde J, Busch AJ, Schachter CL et al. Aerobic exercise training for adults with fibromyalgia. Cochrane Database Syst Rev. 2017;6:CD012700.

12. Bernardy K, Füber N, Klose P, Häuser W. Efficacy of hypnosis/guided imagery in fibromyalgia syndrome--a systematic review and meta-analysis of controlled trials. BMC Musculoskelet Disord. 2011;12:133.

13. Bernardy K, Füber N, Köllner V, Häuser W. Efficacy of cognitive-behavioral therapies in fibromyalgia syndrome - a systematic review and metaanalysis of randomized controlled trials. J Rheumatol. 2010;37:1991-2005.

14. Sarzi-Puttini P, Atzeni F, Salaffi F, Cazzola M, Benucci M, Mease PJ. Multidisciplinary approach to fibromyalgia: what is the teaching. Best Pract Res Clin Rheumatol. 2011;25:311-319.

15. Macfarlane GJ, Kronisch C, Dean LE et al. EULAR revised recommendations for the management of fibromyalgia. Ann Rheum Dis. 2017;76:318-328.

16. Hoffmann TC, Glasziou PP, Boutron I et al. Better reporting of interventions: template for intervention description and replication (TIDieR) checklist and guide. BMJ. 2014;348:g1687.

17. Higgins JPT, Thomas, J, Chandler J et al. Cochrane Handbook for Systematic Reviews of Interventions version 5.1. 2011

18. Moher D, Liberati A, Tetzlaff J, Altman DG, PRISMA G. Preferred reporting items for systematic reviews and meta-analyses: the PRISMA statement. BMJ. 2009;339:b2535.
