# Supplementary material for: Self-management for chronic widespread pain including fibromyalgia: A systematic review and meta-analysis
Source: PLoS One. 2021 Jul 16;16(7):e0254642. doi: 10.1371/journal.pone.0254642 (PMC8284796; doi:10.1371/journal.pone.0254642)
Supplement: S2 File — (PDF) [file pone.0254642.s002.pdf]

MEDLINE search strategy

Ovid MEDLINE(R) Epub Ahead of Print, In-Process & Other Non-Indexed Citations, Ovid MEDLINE(R) Daily and Ovid MEDLINE(R) 1946 to 08 Nov 2017

Search ID#	Query	Items found
1	exp FIBROMYALGIA/	8050
2	fibromyalgia.ti,ab,kf.	9432
3	fibromyositis.ti,ab,kf.	21
4	fibrositis.ti,ab,kf.	618
5	myofascial pain.ti,ab,kf.	1902
6	chronic generalized pain.ti,ab,kf.	19
7	chronic generalised pain.ti,ab,kf.	2
8	chronic widespread pain.ti,ab,kf.	723
9	1 or 2 or 3 or 4 or 5 or 6 or 7 or 8	12855
10	exp Self-Care/	52502
11	(self adj2 (admin$ or care or efficacy or guid$ or help or improve$ or manag$ or monitor$)).ti,ab,kf.	108529
12	Patient Education as Topic/	82679
13	((patient$ or adult$ or client$ or participant$ or individual$) adj2 (train$ or educat$ or teach$ or instruct$ or inform$ or counsel$ or empower$ or advic$ or advis$)).ti,ab,kf.	121485
14	expert patient$.ti,ab,kf.	223
15	Psychoeducation$.ti,ab,kf.	4134
16	(bibliotherapy or book$ or leaflet$ or CD or compact disc$ or DVD or learn$ or guid$ or facilitat$).ti,ab,kf.	1526289
17	exp TELECOMMUNICATIONS/	83971
18	(telemedicine or tele medicine).ti,ab,kf.	9975
19	(telehealth or tele health or tele-health).ti,ab,kf.	3453
20	(ehealth or e-health).ti,ab,kf.	4211
21	(mobile health or mhealth or m-health).ti,ab,kf.	3737
22	ICT.ti,ab,kf.	4364
23	((inform$ or communicat$ or interact$) adj6 (computer$ or technolog$ or software)).ti,ab,kf.	39896
24	((health$ or treat$ or therap$ or intervention$ or assist$ or selfmanag$ or self-manag$) adj6 (computer$ or technolog$ or software)).ti,ab,kf.	91194
25	(interactive voice response or IVR).ti,ab,kf.	1709
26	exp INTERNET/	69683
27	(internet$ or world wide web or www or web or web-based or email$ or e-mail$ or online).ti,ab,kf.	196340
28	(telephone$ or phone$ or mobile$ or cellphone$ or app or apps or text$ or SMS or smartphone$).ti,ab,kf.	305855
29	(virtual reality or augmented reality or VR or AR).ti,ab,kf.	55921
30	10 or 11 or 12 or 13 or 14 or 15 or 16 or 17 or 18 or 19 or 20 or 21 or 22 or 23 or 24 or 25 or 26 or 27 or 28 or 29	2364837
31	9 and 30	1854
