# Supplementary material for: Self-management for chronic widespread pain including fibromyalgia: A systematic review and meta-analysis
Source: PLoS One. 2021 Jul 16;16(7):e0254642. doi: 10.1371/journal.pone.0254642 (PMC8284796; doi:10.1371/journal.pone.0254642)
Supplement: S3 File — (PDF) [file pone.0254642.s003.pdf]

S3 File – Table of characteristics

Study/ Country	Diagnosis/ recruited from	Sample n (int. /controls)	Femal e %	Follow-up assessment points	Multicomponent intervention summary	Control intervention(s)	Intervention duration, delivery & physical setting	Discussion of theory underlying the intervention	Relevant outcome measures
Amris et al. (2014)/ Denmark	Chronic widespread pain (CWP)<br><br>Participants were recruited from the outpatient clinic of a Department of Rheumatology.	96/95	100%	6 months	*Multicomponent treatment course*: Education on CWP, pain, pain management, active self-management of pain and stress, sleep cohabitation, sexuality. Physical therapy including graded exercise activity pacing, aerobic exercise, balance training, relaxation. Occupational therapy, adapting environment to increase participation in activities of daily living.	Waiting list control	Daily sessions for 2 weeks. Group.<br><br>Outpatient.<br><br>Physical setting not clear.	No explicit mention of theory underlying intervention	Physical Function; Pain; FIQ; Quality of life; Depression & Anxiety.
Astin et al. (2003)/ USA	Fibromyalgia<br><br>Participants were recruited from the community by radio and news paper as well as networking with local physicians.	64/64	99%	8 week; 16 weeks; 24 weeks (6 months).	*Mindfulness-based stress reduction (MBSR) with Qigong*. MBSR central focus on learning meditation. Qigong; qigong specific movement/activity.	Education support group: Including stress management, exercise, pain cycle/emotions, sleep, work, intimacy, discussion of current research. Unstructured group discussion.	1 session weekly over 8 weeks. Group.<br><br>Participants were recruited from the community by radio and news paper as well as networking with local physicians.<br><br>Outpatient.<br><br>Physical setting not clear.	Refers to MBSR related constructs: "The goal of this practice is to cultivate a stable and nonreactive awareness of one's internal (e.g., cognitive-affective-sensory) and external (social-environmental) experiences as contrasted with the tendency humans have to react quite reflexively (habitually or automatically) to the myriad situations and experiences (whether stressful or challenging, or not) encountered in daily life." p2258	Physical Function; Pain; FIQ; Depression.
Bosch et al. (2002)/ Spain	Fibromyalgia<br><br>Participants were recruited from a primary care centre.	33/32	100%	4 weeks (post intervention)	*Health education programme:* Fibromyalgia education, discussion of physical-rehabilitative measures and pharmacological treatments,	Control group (no intervention)	1 session weekly for 4 weeks.<br>Group.	No explicit mention of theory underlying intervention	Quality of Life; Pain.

					physical exercise and postural hygiene, relaxation and visualisation.		Outpatient.<br><br>Physical setting not clear.		
Bourgault (2015)/Canada	Fibromyalgia<br><br>Participants were recruited through announcements in local newspapers.	29/29	90%	11 weeks (following intervention end), 3 months after the intervention	*Multicomponent interdisciplinary group intervention:* Each session consisted of psycho-educational tools, CBT-related techniques, and patient-tailored exercise activities.	Waiting list control	8 sessions over 11 weeks, 9th session held at 6 months. Group.<br><br>Outpatient.<br><br>Sessions conducted in an exercise room with PowerPoint facility	No explicit mention of theory underlying intervention.	Pain; FIQ; Global health measure; Quality of life; Depression; Physical function.
Buckelew et al. (1998) / USA	Fibromyalgia<br><br>Participants were referred by personal physicians including rheumatologists and physiatrists at a University Hospital and a private rheumatology practice. Outpatient	30/29/30/30	91%	Post-treatment, 3 months, 1-year, 2-year.	*Combined biofeedback and exercise intervention*. Biofeedback intervention included cognitive and muscular relaxation strategies, and how to apply these strategies to daily living. This aspect of the intervention featured self-monitoring, homework assignments and practice. The exercise intervention comprised range of motion exercises, strengthening exercises and low to moderate intensity aerobic exercise.	1) Biofeedback intervention alone (as described)<br>2) Exercise intervention alone (as described)<br>3) Educational/attention control. This group received educational information about fibromyalgia as well as more general ‘health topics’.	1 session a week for 6 weeks. Individual format. Maintenance phase: 1 session a month. Group.<br><br>Physical setting not clear.	No explicit mention of theory underlying intervention	Physical function; Pain; Global Severity Index (MH measure e.g. depression).
Burckhardt et al. (1994)/ Sweden	Fibromyalgia<br><br>Participants were recruited from occupational health and primary health clinics.	33/31/35	100%	12 weeks	*Self-management education + physical activity*: Education included information on FMS, the role of stress, coping strategies, problem solving techniques, assertiveness training, relaxation strategies, and centrality of physical conditioning. Group discussion. Physical training: included stretching and	1) Self-management education alone (as described).<br><br>2) Waiting list control	6 sessions held over 6 weeks. Group.<br><br>Outpatient.<br><br>Physical setting not clear.	No explicit mention of theory underlying intervention	Physical function; Pain; FIQ & Tender points; Fatigue; Quality of Life; Depression & Anxiety.

					range-of-motion exercise each time, 2 pool therapy sessions, and individual time to develop a physical fitness training program.				
Castel et al. (2013)/ Spain	Fibromyalgia<br><br>Participants were recruited following consultation with a rheumatologist. Outpatient.	81/74	100%	Post-treatment (12 weeks), 3 months, 6 months, 12 months	*Cognitive Behaviour Therapy (CBT) + physical therapy:* CBT included information about FM, theory of pain perception, cognitive restructuring skills training, CBT for primary insomnia, assertiveness training, goal setting, activity pacing, pleasant activity scheduling training, life values, and relapse prevention. Physical therapy focused on aerobic capacity, muscular strengthening, flexibility and alternated with sessions of hydrokinesiotherapy and kinesiotherapy in a gymnasium. All of the sessions included overall aerobic work, coordination exercises, and flexibility exercises. Sessions ended with relaxation. Exercise was encouraged between sessions including a daily walk. CBT and physical therapy were provided in addition to conventional pharmacological treatment.	Conventional pharmacological treatment, adjusted at regular intervals in meeting with doctor	24 sessions held over 12 weeks/ Group.<br><br>Physical setting not clear although states that physical activity conducted in a gymnasium	No explicit mention of theory underlying intervention.	Pain; FIQ; Psychological distress (HADS).
Cedraschi et al. (2004)/ Switzerland	Fibromyalgia	84/80	93%	6 months	*Multidisciplinary self-management programme:* The programme included swimming pool sessions warm water, relaxation exercises, low impact land-based exercises, sessions on activities of daily living, and education-discussion sessions focusing on many aspects of FM such as current scientific knowledge, associated	Waiting list control	12 sessions, twice a week for 6 weeks. Group.<br><br>Participants were recruited from the division of rheumatology at Geneva University	No explicit mention of theory underlying intervention.	Physical function; Pain; FIQ; Psychological well-being (MH).

					conditions, symptoms, modulating factors, and personal relationships.		Hospital. Outpatient. (NC)		
De Souza et al. (2008)/ Country not clear. (Paper in Portuguese, Corresponding Author from Quebec).	Fibromyalgia<br><br>Not clear where patients were recruited from.	30/30	100%	11 weeks (at the end of treatment), 4 months	*Inter-relational school fibromyalgia treatment programme*. Intervention includes educational focus on pain-fatigue-stress-insomnia-pain cycle, discussion about strategies used to manage pain, prescription of an exercise program to be practiced at home (including strengthening, stretching, and moderate walking), relaxation, nutrition and respecting limits.	Waiting list control	9 sessions over 11 weeks. Group. Outpatient.<br><br>Physical setting not clear.	Discusses inter-relational model proposed by communication theory, as basis for the intervention. Also discusses theory focused on interaction in family systems.	Pain.
Giannotti et al. (2014)/ Italy	Fibromyalgia<br><br>Not clear where patients were recruited from.	21/20	Data not provided	1 month (post treatment), 6 months.	*Rehabilitation programme combining education and exercise:* Education included characteristics of FM; information on the symptoms, diagnosis, treatment, physical health, mental health, instructions on how to perform the various proposed exercises. Exercise included a gradual introduction of novel exercises with a progressive increase in intensity. Exercise included stretching, strengthening, active and passive mobilization, spine flexibility and aerobic training, applied on the upper body, trunk, and lower body.	Control group (no intervention).	2 sessions per week for 10 weeks. Group.<br><br>Outpatient.<br><br>Physical setting not clear.	No explicit mention of theory underlying intervention.	Physical function; Pain; FIQ; Fatigue.
Gowans et al. (1999)/ Canada	Fibromyalgia<br><br>Participants were recruited from a large urban tertiary care centre.	23/22	Data not provided (post intervention: 78% female	6 weeks (measured at end of 6-week treatment period)	*Education and exercise:* Education included information on exercise, postural correction, activities of daily living, sleep, relaxation, medication, nutrition, and psychosocial coping strategies.	Waiting list control	2 sessions per week for 6 weeks. Group.<br><br>Outpatient.<br><br>Physical setting not clear.	No explicit mention of theory underlying intervention.	Physical function; Pain; Fatigue; Depression & Anxiety.

					Warm pool exercises; Each class consisted of 20 minutes of walking/jogging/side-stepping/arm exercises against water resistance and 5 minutes of stretching at the beginning and end of each class.				
Hammond et al. (2006)/ UK	Fibromyalgia<br><br>Participants were recruited from a rheumatology outpatient department at one district general hospital.	97/86	Data not provided (at start of treatment following pre-treatment dropout: 90%)	4 months, 8 months.	*Patient education and exercise:* Education included theoretical causes of fibromyalgia, physiological basis of symptoms, the cycle of fibromyalgia symptom perpetuation, and how self-management approaches can improve symptoms. Exercise included: postural training, stretch, and strengthening exercises using light weights; the Tai Chi for Arthritis programme18 to improve balance, cardiovascular and respiratory function, flexibility, muscular strength. Ps were encouraged to establish a home walking programme. Other aspects included activity pacing, sleep hygiene, relaxation (deep breathing, visualization and proprioceptive neuromuscular facilitation methods), problem solving, pain, fatigue and stress management, including managing negative automatic thoughts.	Relaxation: 1-hour classes once a week for 10 weeks Participants were provided with a copy of the Arthritis Research Campaign booklet on fibromyalgia and an explanation of how relaxation assists with fibromyalgia symptoms. Visualisation, deep breathing and a variety of other relaxation methods were included. Participants were encouraged to try these at home but goal-setting and homework programmes were not used.	Patient education and exercise: 1 session per week for 10 weeks. Group.<br><br>Relaxation group: 1-hour classes once a week for 10 weeks. Group.<br><br>Outpatient. Physical settings not clear.	Describes the theoretical foundations of the intervention as social cognitive theory and a cognitive behavioural approach to self-management.	Physical function; Pain; FIQ; Fatigue; Depression & Anxiety.
Hamnes et al. (2012)/ Norway	Fibromyalgia<br><br>Participants were recruited following referral for a week-long self-management	75/72	96%	3 weeks.	Self-management programme: Broad self-management programme included education on living with the condition, and its mechanisms, stress management, relaxation, physical activity in and out of	Waiting list control.	1 week in-patient programme. Group.<br><br>Inpatient. Hospital setting.	Described pedagogical approach, combined with a cognitive behavioural approach to improving self-efficacy.	FIQ; Distress (MH).

	programme a Lillehammer Hospital for Rheumatic Diseases.				a pool, goal prioritisation, healthy eating, trying new activities. e.g. Nordic walking.				
Hsu et al. (2010)/ USA	Fibromyalgia<br><br>Participants were recruited via flyers sent to physicians, local adverts, and presentations at fibromyalgia support group meetings.	24/21	100%	Post intervention and at 6 weeks for those in control arm. 6 months.	*Affective self-awareness programme*. Education included role of biopsychosocial processes in fibromyalgia and associated conditions. Written emotional disclosure consisted of writing about stress and emotions in free-writing prose, unsent letters, and imagined dialogues. Affective awareness techniques included daily CD-guided exercises that encouraged mindfulness-based practices. Support to reengage in physical and leisurely activity and to not allow pain act a barrier to engage in social activities.	Waiting list control.	Following an individual consultation with a physician, 1 session per week for 3 weeks. Group.<br><br>Outpatient.<br><br>Physical setting not clear.	Provides rationale based on models stressing the importance of an internal locus of control for health outcome. Brief mention of theories of emotional and pain.	Physical function; Pain; Fatigue; Mental health.
Kendall et al. (2000)/ Sweden	Fibromyalgia<br><br>Participants were recruited from a Rheumatology Clinic or Pain and Rehabilita-tion Centre, University Hospital, Linkoping.	10/10	100%	7 days post treatment, 6 months, 18 months.	*Body awareness therapy:* Educational lectures on pain, coping, stress, stress reactions, and ways of dealing with both. Exercises focused on a stable relation to the ground, posture, gait and truncal positioning, and the maintenance of balance related to the central axis of the upright body. Breathing emphasised through exercises. Relaxation exercises designed to promote awareness of muscular tension and muscular relaxation.	*The Mensendieck system:* One-to-one guidance on positioning and movement in activated muscle(s). Taught to integrate sensory and cognitive information. Following first session, sessions followed no rigid content schedule but were individually tailored based on the subject's current situation and her experience of the "homework" assigned.	Body awareness therapy: 1 session per week for 21 weeks. Group.<br><br>The Mensendieck system. 18 individual sessions and two group sessions over 20 weeks.<br><br>Outpatient. Physical setting unclear.	Briefly mentions that both intervention stress the importance of an active understanding of bodily movements in order to interpret bodily symptoms.	Physical function; Pain; FIQ; Fatigue; Global health measure; Quality of life.

Supplementary File 3 – Table of characteristics

King et al. (2002)/USA	Fibromyalgia<br><br>Participants were recruited via rheumatologis-ts in the City of Edmonton	35/42/41/34	100%	12 weeks (at the end of the intervention), and 3 months post treatment.	*Combined exercise and self-management education*. Exercise sessions included exercises that were aerobic in nature and included activities such as walking, aquasize (deep and shallow water), or low impact aerobics. Subjects walked outside if the time of year, or weather permitted. The programme was closely based upon the 1990 American College of Sports Medicine (ACSM) recommendations on quantity and quality of exercise for maintaining and developing cardiorespiratory fitness in healthy adults. Nevertheless, clients were instructed to exercise at a level that felt comfortable to them.<br><br>Self-management education included information on potential cause of FM, goal setting focusing on a significant goal for the subject, maximizing energy for personal activities or household chores, pain or fatigue coping strategies, benefits of exercise, evaluating alternative therapies, and barriers to behaviour change. Sessions oriented away from pain and other symptoms and aimed to focus on leading a well-balanced life	1) Exercise alone (as described)<br><br>2) Self-management education alone (as described)<br><br>3) Waiting list control were provided with instruction for basic stretches and 5 coping strategy techniques. They were offered one of the programmes at the end of the follow-up period	Combined exercise and self-management education: 3 exercises classes a week for 12 weeks. 1 self-management education class per week for 12 weeks.<br><br>Exercise: 3 exercise classes per week for 12 weeks.<br><br>Self-management education: 1 class per week for 12 weeks<br><br>All group based.<br><br>Outpatient. Physical setting not clear.<br><br>.	Discusses how self-management principles were taken from social cognitive theory of enhancing self-efficacy.	Physical function; FIQ.
Koulil et al. (2010)/The Netherlands	Fibromyalgia<br><br>Participants were recruited following referral by a rheumatologist	39/45/29/45	Overall figure not provide (G1 = 97%, G2 =	8 weeks (at the end of the intervention), 6 months.	*Pain-avoidance tailored CBT plus exercise*. CBT element was aimed at reducing daily perceived cognitive, behavioural, emotional and social consequences of pain and related symptoms.	1) Waiting list control for pain-avoidance tailored CBT plus exercise<br><br>2) Waiting list control for pain-persistence	2 sessions per week for 8 weeks (exercise directly followed CBT group work). Group.	Described psychological aspects of treatment based on fear avoidance models, include pain avoidance, and models of pain persistence.	Physical function; Pain; FIQ; Fatigue; Negative mood % Anxiety (MH).

	to an outpatient clinic.		89%, G3 = 93%, G4 = 96%)		Particular focus was placed on pain avoidance behaviours. Exercise training targeted increased physical fitness and flexibility. They included aerobic exercises, hydrotherapy or anaerobic exercises.<br><br>*Pain-persistence tailored CBT plus exercise*. CBT element was aimed at reducing daily perceived cognitive, behavioural, emotional and social consequences of pain and related symptoms. Particular focus was placed on regulating and diminishing pain persistence behaviours. Exercise training targeted increased physical fitness and flexibility. They included aerobic exercises, hydrotherapy or anaerobic exercises.	tailored CBT plus exercise	Outpatient.<br><br>Physical setting not clear.		
Kristjansdottir et al. (2013a)/ Norway<br><br>Kristjansdottir et al. (2013b)/ Norway	Chronic Widespread Pain (including fibromyalgia)<br><br>Participants were recruited following referral to a rehabilitation centre in Moss, Norway by a general practitioner or a medical specialist.	70/70	100%	8 weeks (at the end of the intervention), 5 months. 11 months (paper 2013b)	*Inpatient multidimensional rehabilitation plus a smartphone intervention featuring diaries and daily situational feedback*. Rehabilitation included education in pain mechanisms and CBT-based pain management, groups sessions with a focus on motivational interviewing, aerobic exercise (outdoors, in the pool, in the gym), stretching and relaxation.<br><br>Smartphone intervention included a 1-hour face-to-face meeting with a research nurse where goals and values were discussed and the patient received exercises.	Inpatient multi-dimensional rehabilitation alone (as described).	4 week inpatient rehabilitation (groups), followed by a face-to-face session, daily diary and response (weekdays only) via smartphone for 4 weeks. Elements of group, other aspects unclear.<br><br>Participants were recruited following referral to a rehabilitation	Describes theoretical regarding CBT models of catastrophising, Acceptance and Commitment Therapy, Self-Determination theory and the Elaboration Likelihood Model.	Physical function; Pain; FIQ; Fatigue; Global health measure; Depression (MH).

					Patients completed daily dairies on pain related interference and related constructs and diaries included reminders for self-management activities. Patients received daily individualised therapist feedback based on their responses in the diaries.		centre in Moss, Norway by a general practitioner or a medical specialist. Outpatient. NC.		
Kubra et al. (2013)/Turkey **(Abstract only)**	Fibromyalgia	26/24	Not reported	8 Weeks	*Exercise and patient education*. Patients received a home-based exercise programme, and attended patient education meetings	Exercise only. Patients received a home-based exercise programme.	Not directly reported for either delivery, recruitment, or setting.	No explicit mention of theory underlying intervention.	*Does not report on any of pre-specified outcomes*
Lemstra et al. (2005)/ Canada	Fibromyalgia<br><br>Participants were recruited following referral to the trial team from family physicians.	43/36	85%	6 weeks	*Multidisciplinary rehabilitation*. Intervention included group supervised exercise therapy sessions (stretching, aerobic and light weight exercises), behaviour modification for pain and stress management lectures, 1 group education lecture (education on fibromyalgia and general management), 1 group diet lecture and 2 massage therapy sessions (provided as a reward for physical exercise sessions rather than therapeutic benefit).	Waiting list/standard care with family physician.	Session per week details not provided. States 18 exercises sessions, 2 pain and stress management sessions, 1 education session, 1 dietary session occurred over the 6 week education programme. Group.<br><br>Outpatient.<br><br>Physical setting unclear.	No explicit mention of theory underlying intervention.	Physical function; Pain; Medication usage; Depression (MH).
Lera et al. (2009)/Spain	Fibromyalgia<br><br>Participants were recruited from the Fibromyalgia Unit of the Hospital Sant	43/40	100%	4 months (at the end of the intervention), 6 months.	*Medical treatment including self-management course plus CBT*. Intervention included individual appointments with a rheumatologist for clinical and pharmacological management of pain, sleep, muscular problems,	Medical treatment including self-management course alone (as described).	For MT and self-management course, 1-hour session per week for 4 months. Additional CBT	No explicit mention of theory underlying intervention.	Physical function; FIQ, Tender points (DS); Mental health symptoms (MH).

	Joan de Déu in Manresa, Barcelona.				depression/anxiety. Group sessions included physical education (causes of fibromyalgia, correcting bad postural habits). The main focus of the groups was on physical activity including cardiovascular exercises, stretching and restoration including discussion and psychological support. The CBT programme included education on pain processing, mind-body techniques, behavioural activation, planning and goal management, cognitive restructuring, coping skills, psychosocial support.		session occurred before each self-management group session. Group.<br><br>Outpatient. Physical setting not clear		
Lorig et al. (2008)/USA	Fibromyalgia<br><br>Participants were recruited via links to study site placed on established websites, online newsletters and discussion groups.	40/46	Not provided specifically for fibromyalgia patient in trial	6 months, 12 months	*Online arthritis self-management programme*. Intervention included modules on individualised exercise programmes, cognitive strategies including relaxation and self-talk, managing negative emotions, healthy eating, action planning, problem solving. Moderator led online workshops focusing on self-efficacy. Moderated bulletin board.	Usual care control, continued with usual care for fibromyalgia.	Participants were asked to log in to system at least 3 times a week for 6 weeks.<br><br>Outpatient. Online.	No explicit mention of theory underlying intervention.	Physical function; Pain; Fatigue; Global health measure.

Supplementary File 3 – Table of characteristics

Luciano et al. (2011)/ Spain	Fibromyalgia<br><br>Participants were recruited from general practices that referred patients with suspected Fibromyalgia to rheumatologists. If rheumatologists confirmed fibromyalgia, patient was added to recruitment database.	108/108	81%	2 months (at the end of intervention)	*Psychoeducational treatment programme*. Educational aspects of the intervention featured information about typical symptoms and course, potential causes of the illness, current medical and other treatments, the importance of regular exercise. Autogenic training was led targeting immediate physical and mental relaxation, pain relief and stress reduction. Autogenic training also focused on how emotional experiences manifest in the body, and emotional exchange with others in the group. The link between bodily pain and emotions was highlighted.	Usual care control.	9 weekly session over a 2 month period. Group.<br><br>Outpatient. Course took place in the conference room of a general practice.	No explicit mention of theory underlying intervention.	Physical disability; Pain; FIQ; Fatigue; Depression & Anxiety (MH).
Mannerkorpi et al. (2000)/Sweden	Fibromyalgia<br><br>Participants were recruited from primary health care and rheumatology clinics in Goteborg.	37/32	100%	6 months	*Combined exercise and education programme*. Intervention included an exercise programme in a temperate pool. Exercises focused on endurance, flexibility, coordination and relaxation. The education programme introduced strategies to cope with symptoms and encourage physical activity. The main topics of the discussions were symptoms, explanatory theories for long lasting pain and lifestyle modification.	Control group continued with baseline medical treatment.	Exercise sessions: 1 per week for 6 months. Educational programme was delivered over 6 sessions. Group.<br><br>Outpatient. Physical setting unclear.	No explicit mention of theory underlying intervention.	Physical function; Pain; FIQ; Fatigue; Quality of Life; Depression & Anxiety (MH)
Mannerkorpi et al. (2009)/Sweden	Fibromyalgia and Chronic Widespread Pain<br><br>Participants were recruited from primary health care centres in	81/85	100%	20 weeks, 11-12 months	*Combined education and exercise programme*. Educational intervention focused strategies to cope with FM symptoms. Topic included theories of long term pain, pain alleviation, physical activity, stress, relaxation and lifestyle modification. Exercises sessions were carried out in a	Education programme alone (as described).	Education programme consisted of 6 sessions, exercises programme consisted of 20 sessions over 20 weeks. Group.	No explicit mention of theory underlying intervention.	Physical function; Pain; FIQ; Quality of life; Depression & Anxiety (MH).

	western Sweden.				pool, aiming to improve overall function and motivate regular physical activity.		Outpatient. Physical setting unclear.		
Martin et al. (2014)/Spain	Fibromyalgia<br><br>Participants were recruited from patients referred to the pain management unit of the Hospital Galdakao-Usansolo.	82/71	93%	6 months	*'PSYMEPHY' a psychological, medical, educational and physiotherapeutic intervention*.<br>The psychological aspect of the intervention was based on CBT, including cognitive restructuring, behavioural activation breathing and relaxation exercises. The educational aspect focused on the nature of the condition, pain physiology, daily activities, discussion with peers and staff. Exercise training focused on benefits of exercise and stretching routines. Based on activity modification principles.	Usual care (standard pharmacologic care).	6 sessions over 6 weeks/ Group.<br><br>Outpatient. Physical setting unclear.	Described that the intervention was based on the biopsychosocial model.	Pain; FIQ; Fatigue; Anxiety (MH).
McBeth et al. (2012)/UK	Chronic Widespread Pain<br><br>Participants were recruited from general practices in Aberdeen, Scotland.	112/109/112/109	Overall figure not provided (G1 = 69.7%, G2 = 71.4%, G3 = 66.1%, G4 = 70.5%)	6 months, 9 months	*Telephone CBT with exercise*. TCBT included patient centred assessment and formulation, and patient identification of 2-3 goals. Prevision of a CBT for CWP manual. Patients could focus on behavioural activation, cognitive restructuring, and lifestyle changes. Sessions focused on implementation and related problem solving, later sessions focused on relapse prevention. Exercise included an introductory sessions, then 6 monthly fitness instructor-led sessions. The focus was on increasing cardiorespiratory fitness. Participants were encouraged to attend the gym at least twice per week, and on non-gym days engage in everyday activities e.g. walking.	1) Telephone CBT alone (as described)<br>2) Exercise alone (as described)<br>3) Usual care from family physician	TCBT was delivered over 7 weekly sessions with a follow-up sessions at 3 and 6 months. Exercise component was delivered over 6 monthly sessions, and individual engagement between sessions.<br>TCBT: Individual<br>Exercise: Group.<br><br>Outpatient. Exercise took place at a leisure facility. CBT was	No explicit mention of theory underlying intervention	Pain; Harms; Fatigue; Global health measure; Quality of life (breaks into physical functioning and mental functioning)<br>Health care utilisation.

							delivered over the telephone.		
McVeigh et al. (2006)/UK **(*Conference abstract)**	Fibromyalgia	42/44	100%	8 weeks, 20 weeks.	*Pool-based exercise and education programme*.<br>No further description provided.	Usual medical care.	Not directly reported for either delivery, recruitment, or setting.	No explicit mention of theory underlying intervention.	FIQ.
Rooks et al. (2007)/ USA	Fibromyalgia<br><br>Participants were recruited from rheumatology clinics and primary care practices in Boston and surrounding communities.	55/51/50/ 50	100%	16 weeks (at the end of the intervention), 6 months	*Strength, aerobic and flexibility exercises plus fibromyalgia self-help course*.<br>Exercise included aerobic exercise focused on walking, a range of strength-focused resistance exercises, and flexibility training. The fibromyalgia self-help programme included information about the condition and self-management skills, including techniques to accomplish daily activities and management symptoms.	1) Aerobic exercise focused on walking and flexibility exercises alone<br>2) *Strength, aerobic and flexibility exercises alone (as described)*.<br>3) The fibromyalgia self-help programme alone *(as described)*.	Exercise sessions in all groups were held twice weekly for 16 weeks. The self-help programme was 7-sessions held every two weeks.<br>Group.<br><br>Outpatient. Physical setting unclear.	No explicit mention of theory underlying intervention.	Physical function; Pain; FIQ; Fatigue; Quality of life; Depression (MH).
Salaffi et al. (2015)/Italy	Fibromyalgia<br><br>Participants recruited from a database of FM patients.	36/36	93%	12 weeks (at the end of the treatment)	*Multicomponent intervention*.<br>Intervention included aerobic, strength and flexibility exercises. Education included characteristics of fibromyalgia, treatment options, organising daily activities, application of self-control techniques and an opportunity for discussion.	Usual care.	24 sessions held twice per week over 12 weeks.<br>Group.<br><br>Outpatient. Physical setting unclear.	No explicit mention of theory underlying intervention.	Physical function; Medication usage; FIQ; Fatigue.
Saral et al. (2016)/Turkey	Fibromyalgia<br><br>Participants were recruited from referrals to a Department of Physical Medicine and	22/22/22	100%	6 months	*Long-term interdisciplinary treatment*.<br>Intervention included a CBT programme together with exercise training, and an educational programme.	1) Short-term interdisciplinary treatment. Educational items, exercise and brief CBT programme over 2 days.	Long-term interdisciplinary treatment involved a full day of an exercise education programme followed with a	No explicit mention of theory underlying intervention.	Pain; FIQ, Tender points (DS); Fatigue; Quality of Life; Depression (MH). Physical function (SF-36)-physical component

	Rehabilitation in a Hospital.					2) Usual care (continued with treatment as usual).	prescription for weekly exercise, CBT consisted of 1 session a week for 10 weeks. Short-term interdisciplinary treatment was delivered over 2 days.<br><br>Group for both long term and short term.<br><br>Outpatient. Physical setting unclear.		
Stuifbergen et al. (2010)/USA	Fibromyalgia<br><br>Participants were recruited via advertisements in local media.	106/92	100%	2 months (at the end of treatment), 5 months, 8 months	*Lifestyle Counts intervention*. Intervention included the following topic; maximising health when living with fibromyalgia, lifestyle adjustment (e.g. sleep, rest, pacing), exercise and physical activity, eating healthily, stress management and recognising depression, intimacy and person relationships, women's health issues	Attention control. Classroom sessions on topics related to disease management were carefully scripted so that content did not overlap with that presented in the intervention classes e.g. understanding medication, evaluating health information, enhancing memory, health information.	Lifestyle Counts intervention. 1 session a week over 8 weeks. Plus bi-monthly telephone calls for 3 months. Group.<br><br>Attention control. 1 session a week over 8 weeks. Group.<br><br>Outpatient. Physical setting not clear.	Logic model/theory provided for the intervention: Focusing on the development of knowledge and skills will reduce barriers and enhance resources and self-efficacy is expected to result in greater participation in health-promoting behaviours and a more positive quality of life.	Physical function; FIQ; Quality of Life.
Tousignant-Laflamme et al. (2014)/Canada. **(*Confere**	Fibromyalgia<br><br>Participants were recruited from local rheumatologists	37/37	Not reported	3 months	*Education for symptom management*. Intervention included brief group education sessions fostering self-management principles that incorporate 1) patient education, 2)	Waiting list control.	Single 6-hour session. Group.<br><br>Outpatient. Physical setting unclear.	No explicit mention of theory underlying intervention.	FIQ; Global health measure.

**nce abstract)**	and family physicians.				exercises and 3) cognitive-behavioural strategies to improve symptom management.				
Traistaru et al. (2015)/Romania **(*Conference abstract)**	Fibromyalgia	18/18	100%	6 weeks (after treatment)	*6-week home training programme for fibromyalgia*. Intervention included relaxation training – auto training (Schultz), and submaximal aerobic training daily	Control group (no treatment)	Not reported	No explicit mention of theory underlying intervention.	Pain; FIQ; Quality of life.
Vlaeyen et al. (1996)/The Netherlands	Fibromyalgia<br><br>Recruited from a department of rheumatology within a regional general hospital.	49/39/43	88%	6 weeks (after treatment).	*Cognitive-educational treatment*.<br>The cognitive aspect of the intervention featured techniques aimed at decreasing distorted pain attributions and increasing self-efficacy expectations. The educational aspect provided information about psychosocial factors that influence pain, ergonomic principles in daily activities. Each session ended with an exercise, such as swimming or bicycling.	1) Educational treatment alone with group discussion (attention control). Education treatment alone was as described in the multicomponent group. Group discussion regarding parts of a book about pain, listening to music fragments.<br><br>2) Waiting list control	12 sessions spread over 6 weeks.<br>Group.<br><br>Outpatient.<br>Physical setting not clear.	Biopsychosocial or behavioural approach described where pain disability is caused by emotional, cognitive and environmental factors as well as underlying pathology. A cognitive behavioural approach thus, teaches skills to control pain, disability and improve quality of life.	Pain; Fear & Depression (MH).
Williams et al. (2010)/USA	Fibromyalgia<br><br>Participants were recruited via their primary care or specialist care physician.	59/59	95%	6 months	*Web-enhanced behavioural self-management*.<br>Intervention featured educational lectures giving background knowledge about fibromyalgia as a disease state. CBT oriented modules provided educational, behavioural and cognitive skills designed to help with symptom management, and behavioural and cognitive skills designed to facilitate adaptive lifestyle changes for managing fibromyalgia.	Usual care from family physician	Participant were given full access to the web system and told to access it as new issues arose. Fully online.<br><br>Outpatient.<br>Online.	Describes that multiple theoretical models support CBT aspect of approach including: theory of reasoned action, the theory of planned behaviour, social-cognitive theory, control theory, the health beliefs model operant conditioning, and the biopsychosocial model of pain.	Physical function; Pain; Fatigue; Global health measure; Depressed mood & Anxious mood (MH).

Supplementary File 3 – Table of characteristics

Musekamp et al. (2019)/Germany	Fibromyalgia<br><br>Participants were recruited from German inpatient centres.	316/295	Overall figure not provided: G1 = 93.4% G2 = 95.4%	Post treatment (approx. 28 days), 6 months, 12 months	*Multi-model self-management education*. Intervention featured education and discussion of diagnosis and treatment of FMS, coping strategies for pain and for stress, as well as promotion of physical activity. The programme focused on self-management and moving this into everyday life through action planning.	Treatment as usual in this case was in-patient rehabilitation with a focus on education about FMS and coping with pain.	*Multi-model self-management education*. Six 90 minute sessions within 3 week in-patient rehabilitation programme/ Group.<br><br>Treatment as usual, standard inpatient rehabilitation for FMS. Group. Inpatient. Took place within inpatient rehabilitation centres.	No explicit mention of theory underlying intervention.	FIQ; Depression and anxiety measured with the PHQ-4.
Perez-Aranda et al. (2019)/ Spain	Fibromyalgia<br><br>Participants were recruited from the Rheumatology Service of Sant Joande De ´u Hospital (SantBoi de Llobregat, Spain).	75/75/75	Overall figure not provided: G1 = 97.3% G2 = 98.7% G3 = 98.7%	Post treatment (8 weeks), 12 months	*FibroQoL multicomponent intervention:* Includes a focus on psychoeducation on pathophysiology, diagnosis, and management of FMS symptoms. The intervention then moves to a focus on instructions for self-hypnosis to generate a deep state of relaxation.	*Mindfulness-based Stress Reduction MBSR* (single component): Focuses on intensive and structured training in mindfulness to help people relate to their physical and mental condition in more non-judgmental and accepting ways.<br><br>*Treatment as usual:* Standard treatment for FMS.	*FibroQoL multicomponent intervention:* 2-hour session once a week for 8 weeks. Group.<br><br>*Mindfulness-based Stress Reduction MBSR:* 2-hour session once a week for 8 weeks. Group.<br><br>Outpatient. Physical setting not clear.	No explicit mention of theory underlying intervention.	FIQ-R; HADS.

Pereira Pernambuco et al. (2018)/ Brazil	Fibromyalgia<br><br>Participants were recruited through advertisements in local radio, print newspapers and posters displayed in health facilities of Formiga, Minas Gerais, Brazil.	27/31	100%	11 weeks	*Interrelational school of Fibromyalgia*. Intervention focuses on education about pain and coping with pain. Coping strategies, physical activity, symptom mechanism and nutrition are covered in group sessions. Also includes a discussion of pharmacological and non-pharmacological treatments currently used for FMS.	Waiting list control.	*Interrelational school of Fibromyalgia*. One session per week for 11 weeks. Groups.<br><br>Outpatient. Physical setting unclear.	No explicit mention of theory underlying intervention.	FIQ-R

Note. FIQ (-R) = Fibromyalgia Impact Questionnaire (-Revised); FMS = Fibromyalgia Syndrome; HADS = Hospital Anxiety and Depression Scale; CBT = Cognitive Behavioural Therapy; PHQ-4 = Patient Health Questionnaire 4.
