# Supplementary material for: Self-management for chronic widespread pain including fibromyalgia: A systematic review and meta-analysis
Source: PLoS One. 2021 Jul 16;16(7):e0254642. doi: 10.1371/journal.pone.0254642 (PMC8284796; doi:10.1371/journal.pone.0254642)
Supplement: S4 File — (PDF) [file pone.0254642.s004.pdf]

GRADE evidence profile: Self-management interventions vs. no treatment/usual care for chronic widespread pain inc. fibromyalgia

Quality assessment						Summary of findings			
						Number of patients		Effect (SMD/Narrative)	Quality
No of studies	Limitations	Inconsistency	Indirectness	Imprecision	Publication bias	No treatment /usual care	Self-management intervention		
Objective physical function – short term<br><br>4 RCTs	Serious limitations [1]	Serious Inconsistency [2]	No serious indirectness	Serious imprecision [3]	No serious publication bias	110	111	One study reports significantly improved function in SM intervention participants compared to controls. One study reports significant improvements in function in SM intervention group compared to controls in complete case subgroup analysis only. Two studies report some limited evidence of within group improvements in function in SM intervention group contrasted with little within group change in the control.	⊕<br>Very low<br>(rated down for limitations, inconsistency and imprecision)

Objective physical function – long term<br><br>2 RCTs	Serious limitations [1]	No serious inconsistency	No serious indirectness	Serious imprecision [4]	No serious publication bias	104	102	One study reports significant improvements in function in the SM intervention group compared to controls, one study shows significant within group improvements in the SM intervention group contrasted to a lack of within group change in the control group.	⊕⊕<br>Low (rated down for limitations and imprecision)
Self-reported physical function – short term<br><br>9 RCTs	Serious limitations [1]	No serious inconsistency	No serious indirectness	Serious imprecision [5]	No serious publication bias	365	358	SMD: 0.42 (0.20, 0.64). 5 RCTs<br><br>One study reported significant improvements in subjective function in SM intervention compared to controls. Two studies report within group improvements in the SM intervention group only. One study reports no	⊕⊕<br>Low (rated down for limitations and imprecision)

								significant differences between SM intervention and control group. 4 RCTs	
Self-reported physical function – long term<br><br>10 RCTs	Serious limitations [1]	No serious inconsistency	No serious indirectness	Serious imprecision [6]	No serious publication bias	504	486	SMD: 0.36 (0.20, 0.53). 8 RCTs<br><br>Both studies showed no significant difference between SM intervention and control. 2 RCTs.	⊕⊕<br>Low (rated down for limitations and imprecision)
Pain – short term<br><br>12 RCTs	Serious limitations [1]	Serious inconsistency [7]	No serious indirectness	No serious imprecision	No serious publication bias	516	533	SMD: -0.49 (-0.70, -0.27). 6 RCTs.<br><br>Three studies reported significant reductions in pain in SM intervention compared to controls. Three studies reported no significant differences between SM interventions and controls. 6 RCTs.	⊕⊕<br>Low (rated down for limitations and inconsistency)

Pain – long term<br><br>11 RCTs	Serious limitations [1]	Serious inconsistency [7]	No serious indirectness	No serious imprecision	No serious publication bias	514	621	SMD: -0.38 (CI -0.58, -0.19). 9 RCTs<br><br>3 RCTs. Thee showed no significant difference between SM intervention and control.	⊕⊕<br>Low (rated down for limitations and inconsistency)

1 – Studies have unclear risk of bias.
2 – The direction of effect appears to be similar, but findings differ and are presented differently across studies.
3 – Most studies do not provide estimates of effects compared to the control condition.
4 – Range of studies, some providing CIs and some not.
5 – CIs are relatively wide in meta-analysis. Narratively reviewed studies do not provide CIs.
6 – CIs are relatively wide in meta-analysis, and a range of different measures are used for the outcome
7 – Studies in meta-analysis have moderate heterogeneity, narratively reviewed are inconsistent in findings.
