# Supplementary material for: Self-management for chronic widespread pain including fibromyalgia: A systematic review and meta-analysis
Source: PLoS One. 2021 Jul 16;16(7):e0254642. doi: 10.1371/journal.pone.0254642 (PMC8284796; doi:10.1371/journal.pone.0254642)
Supplement: S5 File — (PDF) [file pone.0254642.s005.pdf]

GRADE evidence profile: Self-management interventions vs. active comparison for chronic widespread pain inc. fibromyalgia

Quality assessment						Summary of findings			
						Number of patients		Effect (SMD/Narrative)	Quality
No of studies	Limitations	Inconsistency	Indirectness	Imprecision	Publication bias	No treatment /usual care	Self-management intervention		
Objective physical function – short term<br><br>4 RCTs	Serious limitations [1]	No serious inconstancy	No serious indirectness	Serious imprecision[3]	No serious publication bias	271	210	All 4 RCTs reported no significant differences between SM intervention and active comparison.	⊕⊕<br>Low<br>(rated down for limitations and imprecision)
Objective physical function – long term<br><br>2 RCTs	Serious limitations [1]	Serious inconsistency [2]	No serious indirectness	Serious imprecision [3]	No serious publication bias	149	145	One study reported no significant improvements in function in the SM intervention group compared to an active comparison. One study reported significant within group improvement in the intervention condition alone.	⊕<br>Very low (rated down on limitations, inconsistency, imprecision.)
Self-reported physical	Serious limitations [1]	No serious inconsistency	No serious indirectness	Serious imprecision [4]	No serious publication bias	380	285	SMD: 0.12 (-0.06, 0.30). 5 RCTs.	⊕⊕<br>Low (rated down for limitations and imprecision)

function – short term<br><br>7 RCTs (5 in meta-analysis; 2 narratively reviewed)								One study reported significant improvement function within SM intervention group, and not within the active comparison group, but they were not directly compared. One study reported the SM intervention group and an exercise group showed improvements compared to an active control. 2 RCTs.	
Self-reported physical function – long term<br><br>10 RCTs	Serious limitations [1]	No serious inconsistency	No serious indirectness	Serious imprecision [5]	No serious publication bias<br>.	755	602	SMD: -0.01 (-0.17, 0.16). 6 RCTs<br><br>Three studies showed no difference between SM intervention and active comparison. One study showed within group	⊕⊕<br>Low (rated down for limitations, and imprecision

								improvements in function in the SM intervention and no within group improvements in the active comparison condition. 4 RCTs.	
Pain – short term<br><br>5 RCTs	Serious limitations [1]	No serious inconsistency	No serious indirectness	Serious imprecision [6]	No serious publication bias	283	227	SMD: 0.04 (-0.28, 0.21). 3 RCTs<br><br>One study found that the SM intervention reduced pain compared to an active comparison, one study reported no difference between the SM intervention and active comparison. 2 RCTs.	⊕⊕<br>Low (rated down for limitations and imprecision)
Pain – long term<br><br>8 RCTs	Serious limitations [1]	No serious inconsistency	No serious indirectness	Serious imprecision [6]	No serious publication bias	560	451	SMD: 0.10 (CI -0.41, 0.34). 5 RCTs.<br><br>One study showed no significant difference between SM intervention and	⊕⊕<br>Low (rated down for limitations and imprecision)

								active control. One study showed no within group difference in pain and did not compared groups. One RCT did not directly compare active comparisons with SM intervention, but reported neither the SM intervention or the active comparisons were more effective than usual care in reducing pain in the long term. 3 RCTs.	

1 – Studies have unclear risk of bias.
2 – Rated down for imprecision as findings from 2 trials differ
3 – This has been rated down imprecision as it is not possible to estimate precision from the paper reporting this study.
4 – CIs are relatively wide in meta-analysis, narratively reviewed studies differ.
5 – CIs are relatively wide in meta-analysis, and a range of different measures are used for the outcome.
6 – CIs are relatively wide in meta-analysis.
